# Epigenetic Modulation on Tau Phosphorylation in Alzheimer's Disease

**DOI:** 10.1155/2019/6856327

**Published:** 2019-04-10

**Authors:** Chao-Chao Yu, Tao Jiang, Ao-Fei Yang, Yan-Jun Du, Miao Wu, Li-Hong Kong

**Affiliations:** ^1^Hubei University of Chinese Medicine, Wuhan, Hubei, China; ^2^Hubei Provincial Hospital of TCM, Wuhan, Hubei, China; ^3^Hubei Province Academy of Traditional Chinese Medicine, Wuhan, Hubei, China

## Abstract

Tau hyperphosphorylation is a typical pathological change in Alzheimer's disease (AD) and is involved in the early onset and progression of AD. Epigenetic modification refers to heritable alterations in gene expression that are not caused by direct changes in the DNA sequence of the gene. Epigenetic modifications, such as noncoding RNA regulation, DNA methylation, and histone modification, can directly or indirectly affect the regulation of tau phosphorylation, thereby participating in AD development and progression. This review summarizes the current research progress on the mechanisms of epigenetic modification associated with tau phosphorylation.

## 1. Introduction

Alzheimer's disease (AD), also known as senile dementia, is a common neurodegenerative disorder among the elderly. Mild memory impairment is the primary first sign of AD. As the disease progresses, cognitive functions, such as comprehension, intelligence, emotion, and language proficiency, along with self-care abilities gradually decline in AD patients. Notably, the onset of AD is closely associated with aging [1]. In fact, AD has become one of the major health-threatening disorders among the elderly, having the 6th highest mortality rate in the United States (US) and with a rapidly rising prevalence rate of 1 million new cases per year. It is estimated that there will be 132 million AD patients by the year 2050. As the global population continues to age, AD has become one of the top medical and social concerns worldwide [2]. The pathogenesis of AD is very complex and involves *β*-amyloid protein metabolism disorder and deposition, neurofibrillary tangle (NFT) formation due to abnormal or excessive tau phosphorylation, cholinergic neuron damage, neuroinflammation, abnormal epigenetic modification, intestinal microbiota dysbiosis, abnormal glucose and lipid metabolism, and oxidative stress [3]. The interrelationships between these mechanisms thus create a complex pathogenic network.

Epigenetic modifications refer to heritable alterations in gene expression by means of DNA regulation, RNA methylation, histone modification, and noncoding RNA, which are not caused by changes in the DNA sequence of the gene. Epigenetic modifications can act as a medium between the external environment and the genome. Importantly, environmental changes and stress responses can induce intracellular epigenetic modifications leading to gene transcription or gene silencing [4]. Therefore, understanding the regulatory mechanisms underlying epigenetic modification will provide new strategies for the prevention and treatment of AD. There is currently no clear correlation between early onset AD (5-10% of total AD) or late onset AD and gene mutations [5, 6]. Although the *β*-amyloid cascade hypothesis emphasizes the dominance of senile *β*-amyloid plaques in the pathogenesis of AD [7], many of the *β*-amyloid-targeting drugs developed in recent years have demonstrated poor efficacy and safety in the treatment of AD patients [8]; therefore, the *β*-amyloid theory has been increasingly questioned. Previous studies have demonstrated that the severity and progression of AD are closely associated with the number of NFTs formed and less with the number of senile *β*-amyloid plaques developed [9, 10]. Importantly, paired helical filaments (PHFs), which are formed via tau protein aggregation, are a major component of NFTs [11]. Thus, the ability to target tau protein signaling has become a major goal of drug research and development for AD. In addition, epigenetic modulation on tau phosphorylation is now the primary focus of targeted drug development. Here, we will provide a review on the epigenetic modifications of tau phosphorylation identified in AD.

## 2. Structure and Biological Function of Microtubule-Associated Tau Proteins

**Figure 1:**
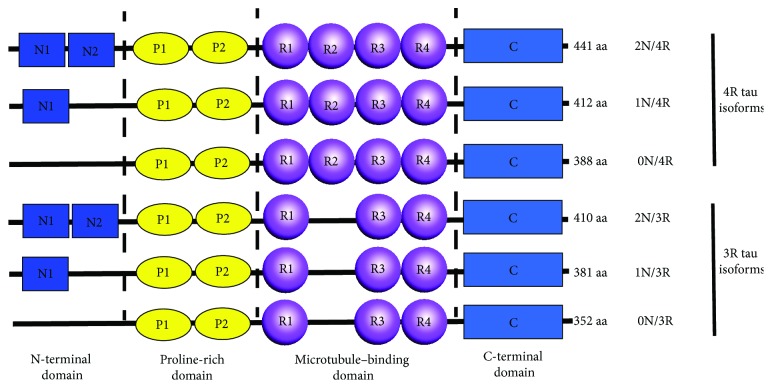
Isoforms of tau protein. The six isoforms of tau are by alternative splicing of exons 2, 3, and 10.

Tau is a microtubule-associated protein (MAP) encoded by the 16 exon-containing *MAPT* gene on chromosome 17 (17q21.31). Tau proteins are rich in neurons in the frontal, temporal, hippocampal, and entorhinal regions of the brain. Intraneuronal tau is predominantly localized in the axons, and it is also present in somatodendritic compartments in much lower levels and contributes to synapse physiology [12, 13]. Markedly, tau proteins bind with a higher affinity to axons than to the cell body or dendrites of a neuron. Depending on the presence or absence of 1 or 2 amino acid insertions in the N-terminus (0N, 1N, and 2N) of the protein and the insertion of either 3 or 4 repeated amino acid sequences in the microtubule-binding domain (3R and 4R) of the protein, a healthy adult human brain can express up to six tau isomers, a result of selective splicing of the *MAPT* gene at exons 2, 3, and 10. The ratio of 4R/3R tau is normally close to 1 : 1 [14, 15]. However, an imbalance in this ratio can lead to neurodegenerative diseases, such as dementia and AD [16, 17]. These tau isomers are comprised of 352, 381, 383, 410, 412, and 411 amino acids and present a molecular weight of approximately 37 to 46 kilodaltons (kDa) [18, 19]. Genetic studies have revealed a relationship between the structural changes in tau and the development of disease pathology. Over 50 mutations in the *MAPT* gene have been identified to date [15], which have been shown to cause abnormal 4R-tau elevation and excessive tau aggregation via interference with the tau protein structure or exon 10 splicing. H1 and H2 are the two haplotypes of *MAPT*. The H1 haplotype is closely associated with an increased risk of late onset AD [20] and Parkinson's disease [21], whereas the H2 haplotype is associated with a reduced risk of late onset AD. The tau protein (Figure 1) is primarily comprised of a N-terminus projection domain, a proline-rich domain, a C-terminus microtubule-binding domain, and a tail domain. The main biological functions of the tau protein include the promotion of microtubule formation, as well as the assembly and promotion of microtubule stability in the cytoskeleton to ensure normal axoplasmic transport and synaptic plasticity [14, 22]. Tau interacts with a large number of partners, thereby acting as the center in cellular protein-protein interaction networks [19]. Interactions between tau and microtubules are mediated by the microtubule-binding repeats and are highly dynamic. The binding of tau to microtubules occurs via 3 or 4 imperfect 18-amino acid repeats (R1–R4) which are located in the microtubule-binding region with a single repeat as the basic microtubule interacting unit [23]. The microtubule-binding region is also involved in the binding of tau to actin filaments and is required for tau aggregation [24]. Several other tau interaction partners have been identified in addition to microtubules. These include membrane-associated proteins such as annexin A2, which contributes to tau's axonal localisation [25]; src-family nonreceptor tyrosine kinases such as Fyn [26], which may be associated with mediating amyloid-beta toxicity at the postsynapse [27]; and protein phosphatase 2A, which serves as the primary tau phosphatase [28]. Evidence indicates that tau oligomers rather than higher aggregates represent the toxic species [29].

The tau protein contains approximately 77 serine/threonine phosphorylation sites. In addition to phosphorylation, other posttranslational modifications on tau including acetylation, methylation, ubiquitination, small ubiquitin-like modifier (SUMO) modification, nitration, glycosylation, truncation, and splicing[14] have been reported and may contribute differentially to physiological functions of tau and disease [30]. In particular, the phosphorylation of tau is the main posttranslational modification event [31]. It should be noted that tau phosphorylation not only just causes damage to neurons but also exerts protective effects. Evidence indicated that tau phosphorylation at the Ser396 and Ser404 sites can render cells antiapoptotic by stabilizing beta-catenin [32]. And tau hyperphosphorylation at the Thr205, Thr231, Ser262, and Ser396 sites can attenuate the endoplasmic reticulum stress- and death-associated protein kinase-induced apoptosis [33, 34]. In addition, tau phosphorylation may also play an important role in adult hippocampal neurogenesis [35]. But when tau is hyperphosphorylated, its affinity for microtubules can be reduced [14]. Hyperphosphorylated tau has been found in the brain of AD patients, with the level of phosphorylation being 3 to 4 times more than that observed in normal individuals [31, 36, 37]. Consequently, tau hyperphosphorylation is currently recognized as an early pathology in AD pathogenesis [38]. The dynamic imbalance between tau phosphorylation and dephosphorylation is mainly caused by abnormal tau protein kinase and protein phosphatase activities. These kinases include glycogen synthase kinase- (GSK-) 3*β*, cyclin AMP- (cAMP-) dependent protein kinase A (PKA), mitogen-activated protein kinase (MAPK), protein kinase (PKC), calmodulin kinase II (CaMK II), microtubule affinity regulating kinase (MARK), and protein phosphatase type 2A (PP2A) [31, 39]. Notably, aberrant tau phosphorylation can lead to microtubule collapse, axon degeneration, and axoplasmic transport disorders, which can subsequently affect neurotransmitter synthesis, transport, release, and uptake, thereby resulting in neurodegeneration [40]. Therefore, the modulation of protein kinases and protein phosphatase activities during tau phosphorylation is currently a major direction of anti-AD drug research and development [41–43].

## 3. Regulation of Tau Phosphorylation by Epigenetic Modification

### 3.1. Noncoding RNA and Tau Phosphorylation

Noncoding RNAs (ncRNAs), including microRNAs (miRNAs), long ncRNAs (lncRNAs), and circular RNAs (circRNAs), are types of non-protein-coding transcription factors that regulate cell function via the regulation of gene expression [44]. There is increasing evidence demonstrating that abnormal ncRNA expression in the brain can affect AD development and progression through multiple molecular pathways [45]. In particular, 20- to 24-nucleotide-long miRNAs are endogenous ncRNAs that have been well studied and are known to play a role in AD pathogenesis. These miRNAs are widely found in the central nervous system (CNS) and play an important regulatory role in neural development, differentiation, and maturation. Furthermore, the miRNA-mediated regulation of target genes is considered a type of posttranscriptional regulation. miRNAs can interact with the 3′ untranslated region (3′-UTR) of the target gene messenger RNAs (mRNAs) via complementary base pairing and induce the degradation or transcriptional suppression of the target mRNA, thereby affecting gene expression. An increasing number of clinical and laboratory studies have now shown that miRNAs play an important regulatory function in the expression of AD-associated genes, including amyloid precursor protein (*APP*), *β*-site APP cleaving enzyme 1 (*BACE1*), *GSK-3β*, and Sirtuin 1 (*SIRT1*) [46]. In addition, miRNAs circulating in the peripheral blood and cerebrospinal fluid (CSF) are also considered potential early diagnostic markers [47] and drug targets [45] for AD. Previous studies have found that many miRNAs, including miR-124, miR-9, miR-132, and miR-137, can alter the 4R/3R tau ratio in neurons by modulating the splicing process of the *MAPT* gene [45].

To this end, a study by Santa-Maria et al. showed that miRNA-219 is downregulated in the brain of AD patients, and subsequent cellular experiments showed that miRNA-219 binds directly to the 3′-UTR of the tau mRNA and represses tau synthesis [48]. Furthermore, miRNA-132 has also been found to be significantly downregulated in AD [49]. miRNA-132 is involved in tau metabolism, as miRNA-132 inhibition can increase amyloid-beta peptide (A*β*) deposition [50] and tau hyperphosphorylation, whereas miRNA-132 upregulation can promote ITPKB and p-ERK1/2 expression, thereby inhibiting tau hyperphosphorylation [51]. It was previously found that the upregulation of miRNA-132 reduced total, phosphorylated, acetylated, and cleaved tau protein levels through the regulation of tau acetyltransferase EP300, GSK-3*β*, RNA binding fox-1 homolog 1 (Rbfox1), calpain 2, and caspases 3/7 protein levels [52]. These changes in turn promote axon extension and bifurcation, enhance synapse plasticity, and prevent neuronal loss. The inhibition of miRNA-132/miRNA-212 can also promote tau protein overexpression, hyperphosphorylation, and aggregation, resulting in cognitive dysfunction [53]. Therefore, given the multitarget properties of miRNA-132, its regulation may be a new potential prevention and treatment strategy for AD [49]. Aside from miRNA-132, many other miRNAs also participate in and influence tau metabolism. For example, the downregulation of brain-derived neurotrophic factors (BDNFs), which are important for the regulation of synapse plasticity, as well as neural growth and differentiation, is closely associated with anxiety and progressive memory loss in AD patients [54]. Evidence indicated that the downregulation of BDNFs is accompanied by the upregulation of miRNA-322 in a mouse model of AD, and further research revealed that miRNA-322 is involved in the phosphorylation of tau proteins via targeted regulation of *BDNF* gene expression and the activity of the TrkB receptor [55]. Binding of BDNF to the TrkB receptor can activate several downstream intracellular signaling cascades including the phosphatidylinositol 3-kinase- (PI3K-) Akt pathway and the Ras-mitogen-activated protein kinase (MAPK) pathway that affect tau phosphorylation [54, 56], while the effects of miRNA-322 on downstream signaling pathways associated with tau phosphorylation such as the PI3K/Akt/GSK-3*β* or MAPK/ERK1/2 pathway remained elusive. Ubiquitin carboxy-terminal hydrolase L1 (UCHL1) is a target of miRNA-922, and inhibition of UCHL1 expression by miRNA-922 promotes tau hyperphosphorylation [57]. Moreover, *in vitro* and *in vivo* experiments demonstrated that miRNA-146a inhibits the expression of the rho-associated, coiled-coil-containing protein kinase 1 (ROCK1) gene and then suppresses tau hyperphosphorylation via ROCK1 regulation through the protein phosphatase and tensin homolog (PTEN) [58]. Evidence has shown that the binding of UCHL1 to PTEN is important for PTEN phosphorylation which promotes tau dephosphorylation [59–61]. In addition, miRNA-12-3p can regulate the expression of the *Caveolin-1* gene and modulate the Caveolin-1-PI3K/AKT/GSK-3*β* signaling pathway to inhibit tau hyperphosphorylation and neuronal apoptosis [62]. Wang et al. reported that the retinoic acid receptor alpha (RARA) is a target gene of miRNA-138 and miRNA-138 can modulate RARA/GSK-3*β* to promote tau hyperphosphorylation [63]. Sun et al. showed that the knockout of miRNA-195 activates Cdk5/p25 signals and promotes the phosphorylation of tau at Ser202, Thr205, Ser262, Thr231, and Ser422 residues. A subsequent study by the authors demonstrated that miRNA-195 can bind to the 3′UTR of the Cdk5r1 mRNA to downregulate the protein expression of p35 and miRNA-195 upregulation in turn suppresses p25 activity, thereby inhibiting tau hyperphosphorylation [64]. miRNA-125b can directly inhibit the expression of Bcl-w to indirectly enhance the activities of tau phosphorylation-associated kinases including Cdk5, p35, and p44/42-MAPK, thus promoting tau hyperphosphorylation [65]. In addition, *in vitro* experiments have demonstrated that members of the miRNA-15 family, such as miRNA-15, miRNA-16, miRNA-195, miRNA-497 [66], and miRNA-26a [67], directly target other tau phosphorylation-associated genes (e.g., *ERK1* and *GSK-3β*) to participate in the development of AD. miRNA-98 is involved in the regulation of tau phosphorylation and *β*-amyloid synthesis via the regulation of the insulin-like growth factor-1 (IGF-1) expression [68]. IGF-1 plays a major role in regulating tau phosphorylation in the aging brain [69], and insulin- or IGF-1-activated PI3K/Akt/GSK-3*β* signaling may be involved in several tauopathies [70, 71]. Evidence showed that the inhibition of tyrosine-protein phosphatase nonreceptor type 1 (PTPN1) can suppress A*β*-induced tau phosphorylation by targeting Akt and GSK-3*β* [72], and PTPN1 was a direct target of miR-124 as validated by the luciferase reporter assay [73]. Rebuilding the miR-124/PTPN1 pathway by suppression of miR-124 or overexpression of PTPN1 restored synaptic dysfunction and memory loss in AD [73]. In addition, Kim et al. identified that the death-associated protein kinase 1 (DAPK1) overexpression increased tau protein stability and phosphorylation at multiple AD-related sites including Ser262, Ser396, and Thr231 [74]. DAPK1 was also a direct target of miR-26a, and miR-26a/DAPK1 signaling cascades were associated with cellular pathologies in neurodegenerative disorders such as Parkinson's disease [75].

Furthermore, Xiong et al. reported that miRNA-218 can modulate GSK-3*β* and phosphatase 2A activities by regulating the expression of the protein tyrosine phosphatase alpha (PTP*α*) [76]. This in turn affects the homeostasis between phosphorylated and dephosphorylated tau proteins. These aforementioned miRNAs were all involved in regulating several signaling pathways which play a significant role in tau phosphorylation.

In addition to those reported miRNAs associated with tau phosphorylation-related signaling pathways, there are also other miRNAs not targeting these pathways but eventually promoting tau hyperphosphorylation. A whole-genome expression analysis indicated that methyl-CpG-binding protein-2 (MeCP2) was a key regulator of tauopathy [77]; a further study confirmed the direct regulation of MeCP2 by miR-132, and the miR-132/MeCP2/dynamin 1 pathway participated in hTau-induced neuronal endocytosis deficiency [78]. In addition, the activation of N-methyl-D-aspartate (NMDA) receptor NR2A, which can also be regulated by miR-125b [79], can decrease tau phosphorylation via the PKC/GSK-3*β* pathway [80]. The protooncogene tyrosine-protein kinase Fyn is a nonreceptor tyrosine kinase primarily expressed in the axons of neurons, which is involved in the regulation of nervous system development and in neuroinflammation, as well as synapse function and neural plasticity [81]. Previous studies have shown that the interaction between tau and Fyn impairs the stability of receptor complexes in the postsynaptic density (PSD) structure and plays an important role in AD pathogenesis [82]. Liu et al. reported that *Fyn* is a target gene of miRNA-106b. Fyn overexpression can promote tau phosphorylation at the Tyr18 site, and miRNA-106b upregulation can inhibit Fyn-induced Tyr18 phosphorylation [83].

Moreover, tau acetylation promotes not only tau autophosphorylation but also abnormal tau aggregation. The acetyltransferase p300 [84] and deacetylase SIRT1 [85] are involved in the regulation of tau acetylation. Reduced SIRT1 levels in the brain of AD patients lead to tau hyperacetylation and consequently tau hyperphosphorylation. Numerous studies have now found that the *SIRT1* gene is directly targeted by miRNA-9, miRNA-212, miRNA-181c, and miRNA-132 [86, 87]. In summary, miRNAs can directly or indirectly (Table 1) affect the expression and activity of several tau phosphorylation-associated proteins and signaling pathways and then modulate tau phosphorylation. Therefore, regulation of these miRNAs may serve as a potential strategy for the development of effective anti-AD therapeutics.

### 3.2. DNA Methylation and Tau Phosphorylation

DNA methylation is a common form of epigenetic modification. This process occurs when the cytosine-guanine dinucleotide (CpG) is modified into 5-methylcytosine by the addition of a methyl group, donated by the S-adenosylmethionine (SAM), on the C5 of CpG in the presence of DNA methyltransferases (i.e., DNMT1, DNMT3A, DNMT3B, and DNMT3L) [90]. The synthesis of SAM is closely associated with vitamin B and folic acid, with CpG being the primary site of DNA methylation. CpG sites are densely found in certain regions of the genome and can be up to 200 base pairs (bp) in length. The CpG island (CpGI) is a region of the genome in which CpG sites comprise 60% to 70% of the sequence and the CpG observed/expected ratio (ObsCpG/ExpCpG) has been shown to be less than (<) 0.6. CpGIs are primarily found upstream of the promoter region/transcription initiation site [91]. The methylation of CpGIs in the promoter region can repress the transcription of target genes and is therefore considered a form of transcriptional regulation [92]. Numerous studies have demonstrated that DNA methylation plays an important role in the aberrant expression of AD-associated genes. Further, immunohistochemical (IHC) analysis of pathological brain tissue sections from deceased AD patients has revealed decreased DNA methylation levels in the prefrontal cortex [93], entorhinal cortex [94], and hippocampus [95]. Moreover, reduced DNA methylation can promote the activation of astrocytes and microglia and proinflammatory cytokine secretion during aging, which consequently results in the vicious cycle of a number of pathological processes [96].

Current clinical and basic research studies have confirmed the presence of abnormal methylation levels in the promoter regions of tau phosphorylation-related genes. For instance, analysis of DNA methylation in the promoter region of the *GSK-3β* gene from the prefrontal cortex tissue of deceased AD patients indicated that the *GSK-3β* promoter region is methylated at low levels during early AD development. The mRNA of GSK-3*β* is upregulated during this period, but the protein expression levels of GSK-3*β* remain unchanged [97]. Some studies have found that vitamin B deficiency can lead to low levels of cytosine methylation in the *GSK-3β* promoter region and hence the GSK-3*β* overexpression [98]. Using chromatin immunoprecipitation (ChIP) and bisulfite sequencing technologies, Li et al. found that the promoter region of Cdk5 has a low level of cytosine methylation in the hippocampal CA1 region of a rat model with A*β*_1-42_-induced memory deficiency. The upregulation of Cdk5 expression leads to tau hyperphosphorylation and suppressed long-term synaptic potentiation, resulting in spatial learning and memory impairment in this rat model [99]. In addition, it was previously reported that AD patients have increased methylation in the promoter region of the *dual-specificity phosphatase 22* (*DUSP22*) gene and downregulated DUSP22 expression, which in turn inhibited PKA-mediated tau phosphorylation and cAMP response element-binding protein (CREB) activation [100] and affected synapse plasticity and long-term memory formation [101]. Besides, DNA demethylation regulated by ten-eleven translocation proteins (Tet1-3) that oxidize 5-methylcytosine (5mC) to 5-hydroxymethylcytosine (5hmC) [102] could also affect tau phosphorylation. Several studies have proved that Tet1 activity functions in active DNA demethylation and gene regulation during learning and memory [103–105]. It is known that BDNF is a key component in the maintenance of synaptic plasticity and synaptogenesis in the hippocampus [56] and is closely related to tau hyperphosphorylation [54, 55, 106]. Ambigapathy et al. reported that Tet1 and ERK1/2 were critical partners regulating BDNF chromatin status and promoter accessibility [107]. It is reasonably assumed that BDNF DNA demethylation regulated by Tet1 could influence the tau phosphorylation levels. These studies demonstrate that targeted regulation of methylation levels of tau phosphorylation-related genes is a potential treatment strategy for AD.

### 3.3. Histone Modifications and Tau Phosphorylation

A nucleosome is the basic unit of DNA packaging, which consists of a segment of DNA wound around histone proteins. H1/H5, H2A, H2B, H3, and H4 are the primary histone proteins important for maintaining the chromosome configuration in DNA material. H2A, H2B, H3, and H4 are core histone proteins, whereas H1/H5 are linker histones. Histones can be modified by acetylation, methylation, phosphorylation, ubiquitination, SUMO modification, and glycosylation. These modifications can affect gene transcription by modulating the spatial conformation of chromatins. For example, acetylation and methylation of lysine (K) and arginine (R) in the N-terminus of histones can neutralize the positive charges on these residues, leading to a reduced affinity between the DNA and histones and loosened chromatin structure (euchromatin) that are favorable for the binding of transcription factors to DNA and hence gene transcription. In contrast, deacetylation of histones tightens the spatial conformation of chromatins (heterochromatin) and suppresses gene transcription [108]. Enzymes that are mainly involved in histone acetylation include histone acetylase (HAT) and histone deacetylase (HDAC). Several studies have recently shown that histone modification plays a role in AD development and progression. It was reported that AD patients and AD mouse models have elevated levels of HADC2 in the brain [109], and inhibition or knockout of HADC2 can significantly improve cognitive dysfunction [110]. Furthermore, both AD patients and the 3xTg and APP_SDI mouse model have an elevated level of Lys12 acetylation on histone H4 (H4K12) as seen in brain tissue samples [111]. Notably, treatment with an HDAC inhibitor can induce hippocampal axonal regeneration, increase axon number, and improve learning and memory in CK-p25 mice [112]. The HADC inhibitor phenylbutyrate can reduce tau hyperphosphorylation, increase axon density, and improve the spatial learning and memory impairment seen in Tg2576 AD mice [113]. In addition, a study by Li et al. showed that increased histone H3 acetylation can lead to tau hyperphosphorylation and impaired synaptic plasticity by promoting Cdk5 transcription and expression [99]. Hippocampal HDAC2 overexpression in 3xTg-AD mice resulted in the deacetylation of the hepatocyte nuclear factor 4 alpha (HNF-4*α*), which allows HNF-4*α* to bind to the promoter of miRNA-101b and repress miRNA-101b expression. This subsequently upregulates AMPK expression and tau hyperphosphorylation, resulting in a reduced density and abnormal morphology of dendrites and consequently cognitive dysfunction in AD mice [114]. Aside from the regulatory role of histone acetylation in tau phosphorylation, Mastroeni et al. also found that an increased Lys4 methylation of histone H3 (H3K4me3) may be involved in tau pathology as an early event in AD pathology. Analysis of brain tissue sections from deceased AD patients revealed that the level of H3K4me3 in the cytoplasm of neurons is elevated as the Braak staging increased [115]. HDAC6 is another important epigenetic component of the etiopathogenesis of AD, and its specific role in AD has been extensively discussed in previous reviews [116, 117]. HDAC6 significantly increased during AD progression [118]. Recent evidence showed that the inhibition of HDAC6 can reverse tau phosphorylation and restore microtubule stability, leading to the normalization of synaptosomal mitochondrial function and synaptic integrity [119, 120]. This evidence indicates that HDAC6 inhibitors may be a promising avenue for therapeutic intervention in AD and other neurodegenerative diseases. However, how HDAC6 impacts genes or signaling cascades related to tau phosphorylation is less reported which warrants further investigation.

## 4. Summary and Future Directions

**Figure 2:**
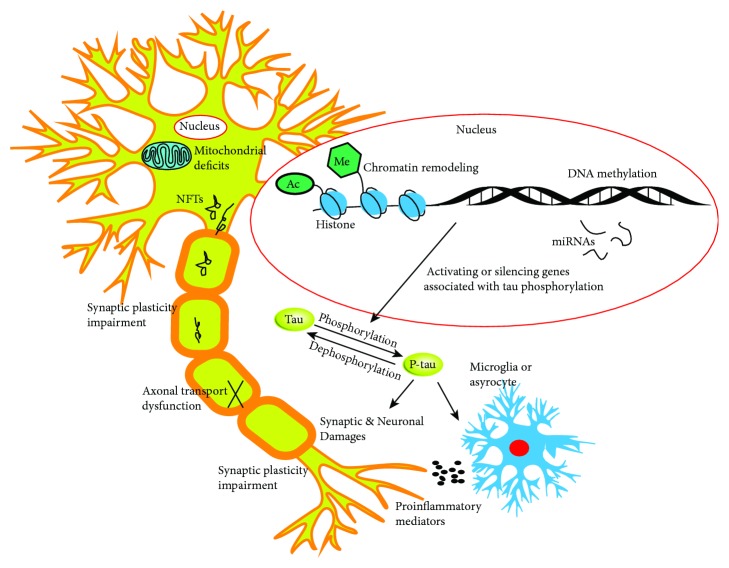
Epigenetic modulation on tau phosphorylation and possible impacts on synapses and neurons. Dysregulated epigenetic modification on genes associated with the tau phosphorylation process could lead to NFT aggregation which can then cause neuroinflammation, axonal transport dysfunction, and mitochondrial and synaptic plasticity injuries.

AD is a pathologically complex neurodegenerative disease, and elucidating the underlying molecular mechanisms of related epigenetic modifications has provided new insights into the understanding of AD pathogenesis, creating new strategies for AD prevention and treatment. As previously mentioned, tau hyperphosphorylation is a key early event in AD pathology, and its development and progression is closely associated with aberrant epigenetic modifications such as miRNAs, DNA methylation, and histone modification. However, whether aberrant epigenetic modifications are the cause or consequence of AD development is still unclear. A review of the mechanisms by which epigenetic modification participates and regulates tau phosphorylation shows that ncRNAs, DNA methylation, and histone modification can directly or indirectly affect the expression and activity of tau-related kinase genes, forming a complex epigenetic regulatory network (Figure 2). Yet, it is still unclear which type of epigenetic modification plays a dominant role in tau hyperphosphorylation, as well as in NFT formation and deposition. This particular point is especially important for the development of specific antitau hyperphosphorylation agents. Therefore, the exact mechanisms by which epigenetic modification participates in tau phosphorylation will need to be further investigated.

Since the different phosphorylation sites of the tau protein can yield different effects which may be protective for neurons instead of causing damage, the potential effects of selected phosphorylation sites modified by epigenetic mechanisms of tau protein kinases and protein phosphatases should be considered. Also, to which degree the epigenetic modulation on tau phosphorylation will be protective should be investigated. So far, no existing evidence mentioned above has taken these issues into account which are important and need to be studied further. Importantly, the occurrence of the altered expression of distinct miRNAs, aberrant DNA methylation, and histone modification involved in tau hyperphosphorylation could be used as new and promising biomarkers for AD in the future. This approach could provide a scientific foundation for the preclinical use of drugs.

There is increasing evidence pointing to the brain stem nucleus as a possible initial site of AD pathology and dissemination. In particular, the raphe nuclei and locus coeruleus may be early regions of NFT formation and aggregation [121–124]. A magnetic resonance imaging (MRI) study revealed that AD patients have altered brain stem volume and morphology [125]. Furthermore, symptoms of cognitive dysfunction in early AD, such as depression and abnormal emotion, in addition to changes in breathing and electrocardiogram (ECG) readings, are closely associated with the secretion of 5-hydroxytryptamine (5-HT) and norepinephrine by the locus coeruleus and raphe nuclei [126–128]. The locus coeruleus is the primary region of norepinephrine synthesis, and noradrenergic neurons in the nuclei project to various regions of the brain in a long-range and extensive manner. Braak et al. showed that NFT formation occurs earlier in the locus coeruleus than in any other brain regions and may be present without any significant clinical symptoms [129]. Neurons in the raphe nuclei are long-range projecting neurons that secrete 5-HT. NFT deposition in the raphe nuclei may explain the abnormal emotional symptoms, such as depression and irritability, that develop during early AD [130] and has also been shown to be associated with the progressive exacerbation of clinical symptoms [131, 132]. Many studies have demonstrated that hyperphosphorylation-mediated tau deposition occurs earlier in the locus coeruleus and raphe nuclei than in other regions of the brain [133] and is often accompanied by neuronal apoptosis [134]. Therefore, early targeted regulation of tau hyperphosphorylation, inhibition of NFT deposition, and formation in the locus coeruleus and raphe nuclei are especially important for the prevention of AD. As key regulators of early AD pathology, epigenetic modifications are also involved in tau hyperphosphorylation in the brainstem nucleus. Andres-Benito et al. found that the neurons in the locus coeruleus in an aging brain presented low katanin-interacting protein gene (*KIAA0566*) methylation levels along with downregulated mRNA and NFT deposition [135]. Researchers have compared the association between the NFT pathology and related miRNA levels in the locus coeruleus, entorhinal cortex, hippocampal CA1 region, and dentate gyrus between Braak stages I and II and stages III and IV and found that miRNA-27a-3p, miRNA-124-3p, and miRNA-143-3p levels in the locus coeruleus, but not any in other regions of the brain, are already elevated in Braak stages I and II and are significantly increased in stages III and IV. Only miRNA-143-3p is elevated in the entorhinal cortex, with all the other miRNA levels remaining unchanged in the hippocampal CA1 region [136]. These findings demonstrate that abnormal epigenetic modification in the locus coeruleus is likely to be involved in the development and progression of tau hyperphosphorylation during early AD pathology. However, the mechanisms by which these epigenetic modifications regulate NFT aggregation in the raphe nuclei are still elusive and will need to be further investigated. Understanding the epigenetic modification mechanisms underlying these AD-related pathologies in associated brain nuclei may provide new insights into the elucidation of AD pathogenesis and the development of actionable drug targets.

However, for now, clinical epigenetics would not be useful as a potential therapeutic strategy to ameliorate AD because epigenetic-based therapy may affect numerous targets due to the lack of locus specificity. DNA methyltransferase inhibitors (DNMTi) and the histone deacetylase inhibitor (HDACi) class are classified as broad reprogrammers because of their large-scale effects on genomic sites [137]. ncRNA-oriented drugs may also yield potential nonspecific off-target effects [138]. Despite these challenges, a range of epigenetic biomarkers for AD diagnosis are in development [139–141]. Integrating all epigenetic aspects and considering epigenetic factors as highly dynamic and interactive players with cellular metabolism by adopting multiomics technologies such as epigenomics, transcriptomics, metabolomics, and proteomics could help in the discovery of novel diagnostic biomarkers or potential drugs for AD.

## Figures and Tables

**Table 1:** MicroRNAs (miRNAs) associated with tau phosphorylation in Alzheimer's disease.

Dysregulated miRNA(s)	Level in AD	Target site(s)	Reference
miRNA-132	Downregulated	ITPKB, MeCP2, GSK-3*β*, and SIRT1	[51, 52, 78, 87, 88]
miRNA-322	Downregulated	BDNF	[55]
miRNA-922	Downregulated	UCHL1	[57]
miRNA-146a	Upregulated	ROCK1	[58]
miRNA-124-3p	Downregulated	Caveolin-1	[62]
miRNA-138	Upregulated	RARA	[63]
miRNA-195	Downregulated	Cdk5r1	[64]
miRNA-125b	Upregulated	Bcl-w, DUSP6, PPP1CA, NMDA, and GSK-3*β*	[65, 79, 89]
miRNA-15	Downregulated	ERK1	[66]
miRNA-98	Upregulated	IGF-1	[68]
miRNA-124	Upregulated	PTPN1	[73]
miRNA-26a	Downregulated	DAPK1	[75]
miRNA-106b	Downregulated	Fyn	[83]
miRNA-218	Upregulated	PTP*α*	[76]
miRNA-212	Downregulated	SIRT1	[86, 87]

ITPKB: inositol 1,4,5-trisphosphate 3-kinase B; MeCP2: methyl-CpG-binding protein-2; GSK-3*β*: glycogen synthase kinase-3*β*; SIRT1: Sirtuin 1; BDNF: brain-derived neurotrophic factor; UCHL1: ubiquitin carboxy-terminal hydrolase L1; ROCK1: rho-associated, coiled-coil-containing protein kinase 1; RARA: retinoic acid receptor alpha; DUSP6: dual-specificity phosphatase 6; PPP1CA: protein phosphatase 1 catalytic subunit alpha isoform; NMDA: N-methyl-D-aspartate; Bcl-w: Bcl-2-like protein 2; ERK1: extracellular-regulated kinase; IGF-1: insulin-like growth factor 1; PTPN1: tyrosine-protein phosphatase nonreceptor type 1; DAPK1: death-associated protein kinase 1; PTP*α*: protein tyrosine phosphatase *α*.
